# Slightly Symptomatic Cerebral Amyloid Angiopathy-Related Inflammation with Spontaneous Remission in Four Months

**DOI:** 10.1155/2019/5308208

**Published:** 2019-07-04

**Authors:** Syuichi Tetsuka, Ritsuo Hashimoto

**Affiliations:** Department of Neurology, International University of Health and Welfare Hospital, 537-3, Iguchi, Nasushiobara, Tochigi 329-2763, Japan

## Abstract

Cerebral amyloid angiopathy-related inflammation (CAA-ri) is a rare variant of CAA with autoimmune inflammation. A 77-year-old female experienced light-headedness during walking and mild ataxic gait without any other objective neuropsychological deficits. Brain magnetic resonance imaging (MRI) revealed an area of abnormal signal and mild parenchymal swelling in the right parietal lobe, indicating vasogenic edema. T2⁎-weighted gradient echo imaging revealed some subcortical microbleeds in the same lesion. Based on the proposed criteria for CAA-ri, she was diagnosed with probable CAA-ri. After 4 months, the spontaneous improvement was noted in the patient's clinical and radiological findings. This report presents a rare and atypical case of CAA-ri in which the diagnosis was established after the patient underwent neuroimaging for only mild neurological symptoms, and the patient's clinical and radiological findings displayed spontaneous improvement. Despite typical and striking MRI findings of CAA-ri, this patient only presented a minimal symptom; this dissociation could highlight the significance of not misinterpreting any new neurological symptoms. Thus, increased availability of MRI and growing awareness of CAA-ri might result in more incidentally diagnosed cases in the future. Furthermore, this case suggests that it would be better to strictly monitor the clinical-radiological findings of patients with probable CAA-ri who only present with minimal symptoms without the initiation of immunosuppressive therapy.

## 1. Introduction

Cerebral amyloid angiopathy (CAA) is an aging-related disease often reported in the elderly population; in this disease, *β*-amyloid (A*β*) peptides are deposited along the walls of small- to medium-sized arteries in the cerebral cortex, causing fibrinoid necrosis of the vessel wall [1]. CAA causes cerebrovascular disorders (microhemorrhages and clinically large intracerebral hemorrhage) and is closely associated with dementia. In some patients with CAA, subacute reversible encephalopathy occurs because of inflammation surrounding the arterial wall where high levels of A*β* peptides are deposited, a condition known as cerebral amyloid angiopathy-related inflammation (CAA-ri). Clinical symptoms of CAA-ri include subacute mental status changes (59%), headaches (35%), focal deficits/seizures (24%), dementia (12%), hallucinations (12%), and stroke-like signs [2]. In the cerebral spinal fluid (CSF), anti-A*β* autoantibodies are reportedly elevated during the acute phase of CAA-ri, which is considered to result from an inflammatory response to A*β* peptides in the cerebrovascular walls [3, 4]. Diagnosis of CAA-ri is facilitated by radiological imaging, particularly magnetic resonance imaging (MRI), thereby circumventing the need of brain biopsy. Furthermore, CAA-ri is considered to be a crucial differential diagnosis of leukoencephalopathy and dementia in the elderly. Here we report the case of a patient with CAA-ri who presented with mild symptoms despite remarkable and typical brain MRI findings.

## 2. Case Report

**Figure 1:**
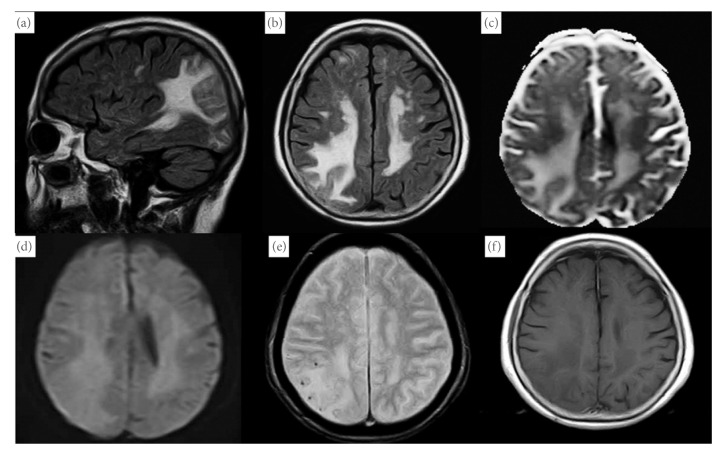
Magnetic resonance imaging (MRI) shows hypersignal intense lesions in the subcortical white matter in the bilateral and asymmetric parietal lobe in both fluid-attenuated inversion recovery (FLAIR) sequence (a and b) and apparent diffusion coefficient (ADC; (c)); however, these lesions were not recognized in diffusion-weighted imaging (DWI; (d)). T2*∗*-weighted gradient echo imaging reveals some subcortical microbleeds in the right parietal lobe (e). Postgadolinium T1-weighted images show no enhancement (f).

**Figure 2:**
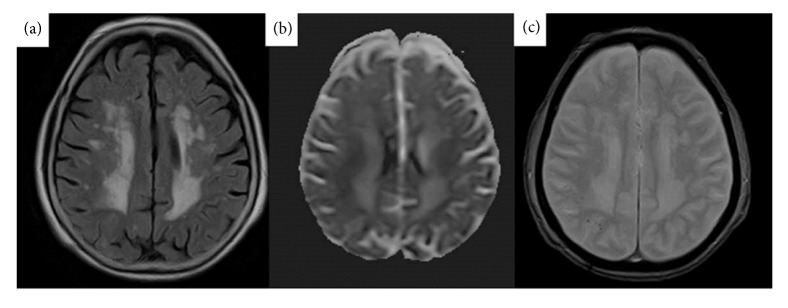
A follow-up brain magnetic resonance imaging (MRI) performed after 4 months shows a reduction of hyperintensity on fluid-attenuated inversion recovery (FLAIR; (a)) and apparent diffusion coefficient (ADC; (b)) in the right parietal lobe. The reduced subcortical microbleeds in the right parietal lobe are noted (c).

A 77-year-old female with hypertension and hyperlipidemia experienced staggering while walking 3 months ago. She fell and bruised her head 2 months ago. At that time, head computed tomography revealed hypodensity in the right parietal region. She presented with a feeling of light-headedness during walking to our hospital. Neurological examination revealed only mild ataxic gait without any other objective neuropsychological deficits. In addition, no cognitive impairment was recognized. Brain MRI revealed an area of abnormal signal and mild parenchymal swelling in the bilateral and asymmetric parietal lobe. Precisely, subcortical hyperintensity was noted in the bilateral and asymmetric parietal lobe on T2-weighted and fluid-attenuated inversion recovery (FLAIR) images and apparent diffusion coefficient (ADC); however, these lesions were not recognized in diffusion-weighted imaging (DWI), indicating vasogenic edema (Figure 1). Moreover, T2*∗*-weighted gradient echo (T2*∗*-GRE) imaging revealed some subcortical microbleeds in the right parietal lobe, and postgadolinium T1-weighted images exhibited no enhancement. Considering her clinical characteristics and MRI findings and excluding the differential diagnosis as infection and brain tumor, she was diagnosed as probable CAA-ri based on the proposed criteria for CAA-ri [5]. We neither performed a brain biopsy to definitively diagnose CAA-ri nor initiated immunosuppressive therapy; instead, we decided to monitor her condition so as to prevent the apparent risks as her clinical symptoms were minimal. During the 4-month follow-up, her symptoms spontaneously disappeared without treatment. After 4 months, brain MRI revealed a reduction in hyperintensity on FLAIR images and reduced subcortical microbleeds in the right parietal lobe (Figure 2). After 12 months following the symptom onset, the patient remains asymptomatic, with stable brain imaging without receiving immunosuppressive therapy.

## 3. Discussion

Here, we present the case of a patient with CAA-ri who was diagnosed after undergoing neuroimaging for mild neurological symptoms; however, this diagnosis was probable based on the proposed criteria [5]. Moreover, the patient's clinical and radiological findings spontaneously improved without immunosuppressive therapy.

CAA implies amyloidosis in the small- and medium-sized blood vessels in the meninges and brain. *β*-amyloid deposits of precursor protein categorize CAA into sporadic and inherited types, with the sporadic A*β*-type CAA accounting for the majority of cases. Although several symptoms are asymptomatic, CAA causes cerebral hemorrhage, cerebral infarction, and transient neurological symptoms, such as abnormal sensation and weakness. Reportedly, sporadic A*β*-type CAA is not a rare disease as its prevalence increases with aging with approximately 30% of autopsy cases of asymptomatic elderly patients exhibiting the sporadic feature [6].

Even more rarely, CAA causes CAA-ri, an inflammatory reaction that is clinically characterized by acute to subacute progressive cognitive dysfunction, headache, positive visual symptoms, coma, seizures, and focal neurological deficits that may be severe [2]. There is an overlap between CAA and Alzheimer's disease (AD), and some CAA patients have associated AD, while many do not. Therefore, when seizures and focal neurological symptoms are observed during the course, it is imperative to consider the probability of AD [7]. However, the patient in our case presented with mild or no symptoms and none of the serious symptoms typical of CAA-ri. To the best of our knowledge, the current findings corroborate only one previous report, till date, of three patients with CAA-ri who presented with only mild or no symptoms [8].

Diagnosis of CAA-ri without invasive brain biopsy is facilitated by MRI, which would typically reveal asymmetrical swelling of the white matter and T2 hyperintense abnormalities, both of which adequately mimic brain tumors, necessitating their clinical differentiation. On T2*∗*-GRE imaging, diagnosis of CAA-ri is facilitated through frequent occurrence of microbleeds below the cortex and subcortex [9]. In our patient, T2*∗*-GRE imaging revealed some punctate low-signal lesions in the cerebral subcortex, and microhemorrhages have remained after the resolution of inflammatory reaction, as previously reported [10]. In central nervous system vasculitis, the lesions appear as hyperintense signal on ADC, suggesting vasogenic edema [11]. As ADC exhibited an intense signal in the white matter lesion in our patient, a similar mechanism can be considered. The bilateral subcortical white matter changes that persist after the disappearance of the right parietal hyperintensity may be attributed to previously existing chronic ischemic changes.

Remarkably, this case presents a dissociation between mild symptoms and notable and typical brain MRI findings of CAA-ri, indicating the significance of not misinterpreting any new neurological symptoms in patients who have already experienced an episode of CAA-ri to receive timely diagnosis and therapy, resulting in better clinical outcome [12, 13]. Had MRI not been performed because of mild or no symptoms in our patient, diagnosing CAA-ri would have been impossible. With the increasing use of MRI, such cases might continue to surge in the future. In addition, the dissociation noted in our patient corroborates a prior report presenting amyloid-related imaging abnormalities (ARIA) [14]. ARIA signifies MRI abnormalities when represented as a result of the major adverse effect of A*β* immunotherapy for AD and represents vasogenic edema and/or sulcal effusion on FLAIR sequences, as well as hemorrhagic findings [14, 15]. Reportedly, CSF anti-A*β* autoantibodies play a pivotal role in the etiopathogenesis of ARIA, and CAA-ri is broadly accepted as a human spontaneous model of the therapeutic-induced ARIA [3, 4, 10]. However, CAA-ri is typically related to marked neurological disturbances, whereas ARIA is often asymptomatic or mild, but always accompanied by dementia [16]. In our patient, although neurological disturbances were asymptomatic or mild like ARIA, cognitive impairment, such as dementia, was not determined at all. Hence, this case is not of spontaneous ARIA, which has been recently identified in AD [17]. Perhaps, it could be diagnosed as CAA-ri that suggests asymptomatic or mild neurological disturbances. Remarkably, both spontaneous ARIA and CAA-ri have also been reported in familial forms of AD [10, 18, 19]. Thus, this case also highlights the necessity of monitoring the disease course in the future.

Remarkably, both clinical and radiological findings demonstrated spontaneous improvement in our patient. Typically, patients with CAA-ri are highly responsive to immunosuppressive therapies, including corticosteroids, or others such as methotrexate, mycophenolate mofetil, cyclophosphamide, and intravenous immunoglobulin if promptly diagnosed and medicated [20–22]. Although the autoimmune mechanism of inflammation in patients with CAA-ri is speculated to be an autoimmune reaction against A*β* peptides resulting in the formation of A*β* autoantibodies in the cerebrovascular walls, the underlying cause of this autoimmune response remains unclear [4]. However, we hypothesize that a spontaneous change in a patient with CAA-ri may occur, which is the movement of anti-A*β* autoantibodies from the vasculature to the brain parenchyma. Antibody binding to vascular A*β* peptides triggers inflammation by trapping A*β* during efflux from the brain. Consequently, it becomes increasingly vulnerable to blood–brain barrier (BBB) damage, microhemorrhage, and vasogenic edema [23, 24]. The empiric treatment with steroids could be reasonable in the clinical setting that fulfills the diagnostic criteria for probable CAA-ri without invasive brain biopsy. Nevertheless, in patients presenting with mild or no symptoms, CAA-ri might spontaneously recover in the future as noted in our patient. Thus, it would be better to carefully monitor probable CAA-ri that only presents with minimal symptoms without initiating immunosuppressive therapy. In addition, spontaneous improvement has been previously reported [25]. Likewise, our patients also did not need above-mentioned immunosuppressive therapies, and spontaneous improvement was noted in both clinical and radiological findings. Moreover, to the best of our knowledge, CAA-ri presenting with minimal or no symptoms and spontaneously resolving without immunosuppression has not been reported till date. Although the reason remains unclear, in our patient, it could be the possible occurrence of CAA-ri spontaneous remission during follow-up similar to other autoimmune diseases. Although patients with CAA-ri tend to be typically highly responsive to immunosuppressive therapy in case of prompt diagnosis and medication and the occurrence of successive relapses is considered rare [12], it is imperative to carefully monitor these patients in the future.

## 4. Conclusions

This report describes a rare and atypical case of CAA-ri, in which patient was diagnosed after undergoing neuroimaging because of only mild neurological symptoms, and the patient's clinical and radiological findings showed spontaneous improvement without immunosuppressive therapy. Despite typical and striking MRI findings of CAA-ri, this patient only presented a minimal symptom; this dissociation between mild symptoms and remarkable and typical brain MRI findings of CAA-ri highlights the significance of not misinterpreting any new neurological symptoms. Thus, increased availability of MRI and growing awareness of CAA-ri might result in more incidentally diagnosed cases in the future. Furthermore, this case suggests that it would be better to strictly monitor the clinical-radiological findings in patients with probable CAA-ri who only present with minimal symptoms without the initiation of immunosuppressive therapy.

## References

[1] Charidimou A., Gang Q., Werring D. J. (2012). Sporadic cerebral amyloid angiopathy revisited: recent insights into pathophysiology and clinical spectrum. *Journal of Neurology, Neurosurgery & Psychiatry*.

[2] Scolding N. J., Joseph F., Kirby P. A. (2005). A*β*-related angiitis: primary angiitis of the central nervous system associated with cerebral amyloid angiopathy. *Brain*.

[3] Difrancesco J. C., Brioschi M., Brighina L. (2011). Anti-A*β* autoantibodies in the CSF of a patient with Caa-related inflammation: a case report. *Neurology*.

[4] Piazza F., Greenberg S. M., Savoiardo M. (2013). Anti-amyloid *β* autoantibodies in cerebral amyloid angiopathy-related inflammation: implications for amyloid-modifying therapies. *Annals of Neurology*.

[5] Auriel E., Charidimou A., Gurol M. E. (2016). Validation of clinicoradiological criteria for the diagnosis of cerebral amyloid angiopathy–related inflammation. *JAMA Neurology*.

[6] Esiri M. M., Wilcock G. K. (1986). Cerebral amyloid angiopathy in dementia and old age.. *Journal of Neurology, Neurosurgery & Psychiatry*.

[7] Castro Caldas A., Silva C., Albuquerque L., Pimentel J., Silva V., Ferro J. M. (2015). Cerebral amyloid angiopathy associated with inflammation: report of 3 cases and systematic review. *Journal of Stroke and Cerebrovascular Diseases*.

[8] Banerjee G., Alvares D., Bowen J., Adams M. E., Werring D. J. (2018). Minimally symptomatic cerebral amyloid angiopathy-related inflammation: three descriptive case reports. *Journal of Neurology, Neurosurgery & Psychiatry*.

[9] Eng J. A., Frosch M. P., Choi K., Rebeck G. W., Greenberg S. M. (2004). Clinical manifestations of cerebral amyloid angiopathy-related inflammation. *Annals of Neurology*.

[10] Boncoraglio G. B., Piazza F., Savoiardo M. (2015). Prodromal alzheimer's disease presenting as cerebral amyloid angiopathy-related inflammation with spontaneous amyloid-related imaging abnormalities and high cerebrospinal fluid anti-A*β* autoantibodies. *Journal of Alzheimer's Disease*.

[11] White M. L., Hadley W. L., Zhang Y., Dogar M. A. (2007). Analysis of central nervous system vasculitis with diffusion-weighted imaging and apparent diffusion coefficient mapping of the normal-appearing brain. *American Journal of Neuroradiology*.

[12] DiFrancesco J. C., Touat M., Caulo M. (2015). Recurrence of cerebral amyloid angiopathy-related inflammation: a report of two cases from the iCA*β* international network. *Journal of Alzheimer's Disease*.

[13] Savoiardo M., Erbetta A., Di Francesco J. (2011). Cerebral amyloid angiopathy-related inflammation: an emerging disease. *The Neuroradiology Journal*.

[14] Di Francesco J. C., Longoni M., Piazza F. (2015). Anti-A*β* autoantibodies in amyloid related imaging abnormalities (ARIA): candidate biomarker for immunotherapy in alzheimer's disease and cerebral amyloid angiopathy. *Frontiers in Neurology*.

[15] Sperling R. A., Jack C. R., Black S. E. (2011). Amyloid-related imaging abnormalities in amyloid-modifying therapeutic trials: recommendations from the alzheimer’s association research roundtable workgroup. *Alzheimer's & Dementia*.

[16] Sevigny J., Chiao P., Bussière T. (2016). The antibody aducanumab reduces A*β* plaques in Alzheimer’s disease. *Nature*.

[17] Carlson C., Estergard W., Oh J. (2011). Prevalence of asymptomatic vasogenic edema in pretreatment Alzheimer's disease study cohorts from phase 3 trials of semagacestat and solanezumab. *Alzheimer’s & Dementia*.

[18] Ryan N. S., Biessels G., Kim L. (2015). Genetic determinants of white matter hyperintensities and amyloid angiopathy in familial Alzheimer's disease. *Neurobiology of Aging*.

[19] Floris G., Di Stefano F., Cherchi M. V., Costa G., Marrosu F., Marrosu M. G. (2015). Multiple spontaneous cerebral microbleeds and leukoencephalopathy in psen1-associated familial alzheimer's disease: mirror of cerebral amyloid angiopathy?. *Journal of Alzheimer's Disease*.

[20] Cenina A. R. F., De Leon J., Tay K. Y., Wong C. F., Kandiah N. (2015). Cerebral amyloid angiopathy-related inflammation presenting with rapidly progressive dementia, responsive to IVIg. *Alzheimer Disease & Associated Disorders*.

[21] Brotman D. J., Eberhart C. G., Burger P. C., McArthur J. C., Hellmann D. B. (2000). Primary angiitis of the central nervous system and Alzheimer's disease: clinically and pathologically evident in a single patient. *The Journal of Rheumatology*.

[22] Greenberg S. M., Parisi J. E., Keegan B. M. (2007). A 63-year-old man with headaches and behavioral deterioration. *Neurology*.

[23] Kaufer D., Gandy S. (2009). APOE 4 and bapineuzumab: infusing pharmacogenomics into Alzheimer disease therapeutics. *Neurology*.

[24] Racke M. M., Boone L. I., Hepburn D. L. (2005). Exacerbation of cerebral amyloid angiopathy-associated microhemorrhage in amyloid precursor protein transgenic mice by immunotherapy is dependent on antibody recognition of deposited forms of amyloid *β*. *The Journal of Neuroscience*.

[25] Liang J. W., Zhang W., Sarlin J., Boniece I. (2015). Case of cerebral amyloid angiopathy-related inflammation – is the absence of cerebral microbleeds a good prognostic sign?. *Journal of Stroke and Cerebrovascular Diseases*.

